# Hemochromatosis in an Adult Female With Previous Iron Deficiency Anemia on Iron Supplementation

**DOI:** 10.7759/cureus.50166

**Published:** 2023-12-08

**Authors:** Keval Yerigeri

**Affiliations:** 1 Internal Medicine-Pediatrics, Case Western Reserve University School of Medicine, Cleveland, USA

**Keywords:** follow-up recommendations, bronze diabetes, iron overload cardiomyopathy, iron deficiency anemia, hemochromatosis

## Abstract

A 56-year-old female presented with a diagnosis of iron deficiency anemia over three years prior (on oral iron supplementation) and presented with altered mental status. She was admitted to the Coronary Care Unit for troponinemia and T-wave inversions. Two-dimensional echocardiography revealed hypokinesia of the left ventricle (LV) anterior wall with reduced ejection fraction. The patient was stabilized on metoprolol and heparin infusions, but heparin was discontinued after iron studies revealed overload (iron: 159 ug/dL, 100% saturation, ferritin: 1480 ng/mL). Cardiac MRI revealed mixed concentric-eccentric LV hypertrophy, raising concern for severe iron overload. Mutation analysis of the *HFE * (homeostatic iron regulator) gene responsible for hereditary hemochromatosis was negative for a homozygous mutation.

Hematology was consulted to establish outpatient follow-up and consider treatments for the acquired hemochromatosis. Acquired hemochromatosis typically occurs in the setting of multiple blood product transfusions. Oral supplementation is typically mitigated by limited bioavailability. Follow-up was needed to track the patient’s iron deficiency anemia and identify resolution and potential toxicity. Further research into predisposing factors for hemochromatosis in patients on oral supplementation without *HFE* mutations is indicated.

## Introduction

This study explores an unexpected case of acquired “bronze diabetes” and iron overload cardiomyopathy in a patient diagnosed with iron deficiency anemia three years before oral iron supplementation. Iron deficiency is a common pathology in women of childbearing age, affecting 10%-15% in Europe and 30% worldwide. It becomes clinically relevant when iron levels decline to levels insufficient for erythropoiesis, precipitating iron deficiency anemia (IDA) [1]. The condition is diagnosed by "iron studies," including serum iron and ferritin levels, total iron-binding capacity, and transferring saturation. Typical management of IDA is oral supplementation with 325 mg daily of ferrous sulfate or ferrous fumarate. On rare occasions, long-term consumption of ferrous products can lead to secondary iron overload, but cases typically involve misdiagnosis or inappropriate, non-prescription use [2]. We present a case of secondary overload in the absence of misdiagnosis, phlebotomy, or HFE mutation homozygosity (responsible for hereditary hemochromatosis).

## Case presentation

A 56-year-old female presented to the emergency department with altered mental status. Past medical history included hypertension, hyperlipidemia, diabetes, hypothyroidism, gastroesophageal reflux disease, and alcoholic cirrhosis complicated by esophageal varices and encephalopathy. She was also incidentally diagnosed with iron deficiency anemia 3-4 years prior during an inpatient admission for jaundice. A work-up for normocytic anemia at the time yielded the iron studies shown in Table 1.

**Table 1:** Results of iron studies 3-4 years before presentation and admission.

Component	Result	Reference Range
Iron	37 ug/dL	45-160 ug/dL
Iron saturation	9%	20%-55%
Total iron-binding capacity	391 ug/mL	250-410 ug/mL
Ferritin	11.1 ng/mL	10.3-219 ng/mL

The patient was started on iron supplementation with a twice-daily regimen of 325 mg ferrous sulfate. She had no subsequent follow-up with hematology or trending iron studies.

Upon arrival at the emergency department, lab findings were pertinent for thrombocytopenia, hyperbilirubinemia, and an elevated prothrombin time and international normalized ratio. The patient was admitted to the Coronary Care Unit for troponinemia (peak 337 ng/L; reference range: 0-10 ng/L). An electrocardiogram (EKG) demonstrated new T-wave inversions thought to be indicative of a type II myocardial infarction. The echocardiogram revealed hypokinesia of the left ventricle anterior wall, with the ejection fraction reduced to 45% +/-5%. Right ventricular function was also globally reduced, and fibrocalcific changes were appreciated in the aortic valve and mitral annulus. The patient was started on a metoprolol infusion for atrial fibrillation with rapid ventricular response on telemetry and a heparin drip for thrombosis prophylaxis. However, anticoagulation was swiftly discontinued upon repeat iron studies shown in Table 2.

**Table 2:** Repeat iron studies on the first day of inpatient admission.

Component	Result	Reference Range
Iron	159 ug/dL	45-160 ug/dL
Iron saturation	100%	20%-55%
Total iron-binding capacity	160 ug/mL	250-410 ug/mL
Ferritin	1480 ng/mL	10.3-219 ng/mL

**Figure 1:**
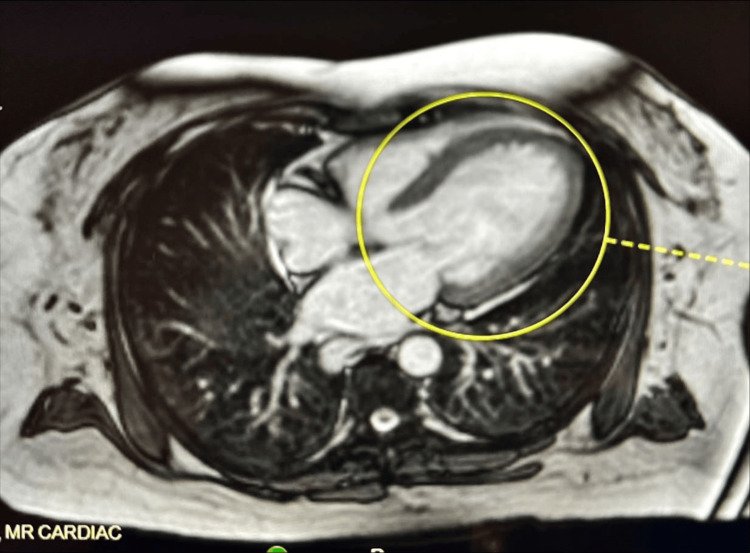
Cardiac MRI from day three of admission; axial true fast imaging with steady-state free precession of the whole chest (flip angle 80°). The left ventricle is encircled in yellow. Circle: Mixed concentric and eccentric left ventricular hypertrophy was appreciated

Given the concern for iron overload affecting cardiac physiology, a cardiac MRI was ordered inpatient. Very low T2 values in the heart raised concern for severe iron overload. Mixed concentric and eccentric left ventricular hypertrophy was appreciated (circled in Figure 1), and left ventricular systolic function was severely reduced to 29% (likely due to myocardial iron deposition). The right ventricular size was normal, but systolic function was mildly reduced, with an ejection fraction of 46%.

Of note, hemoglobin A1c one month before the admission was elevated to 8.4% versus 5.7% from two years prior. The family of the patient also remarked that the patient’s skin was distinctly discolored to a darker hue, raising concern for “bronze diabetes” in the primary team. HFE gene mutation analysis identified one copy of the H63D pathogenic variant (consistent with a carrier phenotype) and was negative for the C282Y variant. These results reduced the likelihood of hereditary hemochromatosis but could not rule out other pathogenic variants. The patient has also denied any transfusions for variceal bleeding or otherwise in the past three years. Hematology was consulted to establish outpatient follow-up for the management of supposed acquired hemochromatosis secondary to oral iron supplementation. The ferrous sulfate tablets and anticoagulation were held upon discharge. Hematology follow-up was scheduled with consideration of phlebotomy versus chelation therapy.

## Discussion

Iron is integral to several life processes, including oxygen transport and energy facilitation [3]. Hemoglobin, responsible for oxygen transport from the lungs to peripheral tissues, and myoglobin, an oxygen storage protein in muscle tissues, both include the iron-containing compound heme. Over 95% of functional iron in the body is deployed within heme molecules [4]. Dysregulation of iron metabolism plays a role in several diseases. Notably, hemochromatosis is a disorder of iron overload that can lead to multi-organ failure and death if uncontrolled. It is a common inherited disorder with an incidence of 1 in 200 amongst those with northern European ancestry [5]. Age of onset is typically past 50 in men and post-menopause in women, given iron losses in menses, pregnancy, and breastfeeding. The disease was first coined by pathologist von Recklinghausen in 1889 as a clinical syndrome incorporating cirrhosis, diabetes, and hyperpigmentation (as seen in our patient) [6]. The etiology can be inherited or acquired, as shown in Table 3 [7].

**Table 3:** Inherited and acquired causes of hemochromatosis.

Primary Iron Overload (Hereditary)	Secondary Iron Overload (Acquired)
HFE C282Y homozygosity	Dietary iron overload
HFE C282Y/H63D heterozygosity	Long-term hemodialysis
SLC11A3 gene mutations	Chronic liver disease (eg, alcoholic cirrhosis)
Transferrin receptor 2 gene mutations	Porphyria cutanea tarda

Hereditary hemochromatosis is classified into four types based on genetic etiology. All, save for type 4, are inherited in an autosomal recessive fashion. Type 1 (HFE-related) is the classic form with worldwide prevalence. HFE mutation analysis is commonly tested in the work-up for hemochromatosis (as shown in this case), but the presence of a mutation is not diagnostic. Type 2 is due to mutations of the hemojuvelin or hepcidin genes; onset is typically at 15 to 20 years of age. Type 3 is a mutation of the transferrin receptor-2 gene, with onset at 30 to 40 years of age. Type 4 follows an autosomal dominant pattern involving mutations in the ferroportin gene; the age of onset is highly variable, suggesting a penetrance-based pattern.

The gold standard for hemochromatosis diagnosis is iron studies supported by histologic evidence of iron overload in end-organ systems (e.g., heart, liver, pancreas, pituitary gland). Iron deposits, such as hemosiderin and yellow granules found in the cytoplasm, lead to free radical damage. Characteristic manifestations include liver fibrosis and cirrhosis, bronze hyperpigmentation, diabetes mellitus, malabsorption and fatty stools, cardiomyopathy, secondary hypogonadism, and chondrocalcinosis. Our patient case exhibited several of these findings, including those of the skin, heart, and endocrine systems.

Acquired hemochromatosis is typically due to repeated blood transfusions and the resulting iron overload. Conditions that may warrant this level of blood product support include sickle cell anemia, thalassemia, aplastic anemia, and cancer patients on aggressive myelosuppressive therapies. Each unit of transfused blood contains over 200 mg of iron, one hundred times the recommended daily dietary doses [8]. Oral supplementation does not typically lead to overload due to its limited bioavailability. For a mixed diet, bioavailability is less than 20% in subjects without iron stores [9]. Only 10%-15% of ferrous sulfate is absorbed, albeit at a significantly higher rate than other ferrous compounds.

## Conclusions

Nutritional iron overload takes years to develop and is uncommon without genetic factors or a significant blood transfusion burden, both of which are lacking in our patient case. The suspicion, instead, is that the iron deficiency anemia was overestimated in the context of a low-normal ferritin and high-normal total iron-binding capacity. This patient needed follow-up with a primary care provider or subspecialist to ensure compliance with medications and the recovery of iron stores, not to mention potential toxicity. Hemochromatosis had already contributed to heart failure, poor glycemic control, and the exacerbation of liver disease by the time of presentation. Further research into potential predisposing factors for hemochromatosis in patients on oral iron supplementation without HFE mutations is indicated.
